# Association of phenylalanine and tyrosine metabolism with mortality and response to nutritional support among patients at nutritional risk: a secondary analysis of the randomized clinical trial EFFORT

**DOI:** 10.3389/fnut.2024.1451081

**Published:** 2024-11-12

**Authors:** Lena C. Buchmueller, Carla Wunderle, Rahel Laager, Luca Bernasconi, Peter J. Neyer, Pascal Tribolet, Zeno Stanga, Beat Mueller, Philipp Schuetz

**Affiliations:** ^1^Medical University Department, Division of General Internal and Emergency Medicine, Division of Endocrinology, Diabetes, and Metabolism, Kantonsspital Aarau, Aarau, Switzerland; ^2^Medical Faculty, University of Basel, Basel, Switzerland; ^3^University Hospital of Child and Adolescent Psychiatry and Psychotherapy, University of Bern, Bern, Switzerland; ^4^Institute of Laboratory Medicine, Kantonsspital Aarau, Aarau, Switzerland; ^5^Department of Health Professions, Bern University of Applied Sciences, Bern, Switzerland; ^6^Faculty of Life Sciences, University of Vienna, Vienna, Austria; ^7^Division of Diabetes, Endocrinology, Nutritional Medicine, and Metabolism, Inselspital, Bern University Hospital, University of Bern, Bern, Switzerland; ^8^Faculty of Biomedical Sciences, Università della Svizzera italiana (USI), Lugano, Switzerland

**Keywords:** malnutrition, polymorbid patient, individualized nutrition support, tyrosine, phenylalanine, biomarker

## Abstract

**Background:**

Elevated phenylalanine serum level is a surrogate marker of whole-body proteolysis and has been associated with increased mortality in critically ill patients. Tyrosine is a metabolite of phenylalanine and serves as a precursor of thyroid hormones and catecholamines with important functions in the oxidative stress response among others. Herein, we examined the prognostic significance of phenylalanine, tyrosine, as well as its metabolites nitrotyrosine, L-3,4-dihydroxyphenylalanine (DOPA), and dopamine regarding clinical outcomes and response to nutritional therapy in patients at nutritional risk.

**Methods:**

This is a secondary analysis of the Effect of Early Nutritional Support on Frailty, Functional Outcomes, and Recovery of Malnourished Medical Inpatients Trial (EFFORT), a randomized controlled trial investigating individualized nutritional support compared to standard care in patients at risk of malnutrition. The primary outcome was 30-day all-cause mortality.

**Results:**

We analyzed data of 238 patients and found a significant association between low plasma levels of phenylalanine [adjusted HR 2.27 (95% CI 1.29 to 3.00)] and tyrosine [adjusted HR 1.91 (95% CI 1.11 to 3.28)] with increased 30-day mortality. This association persisted over a longer period, extending to 5 years. Additionally, trends indicated elevated mortality rates among patients with low nitrotyrosine and high DOPA and dopamine levels. Patients with high tyrosine levels showed a more pronounced response to nutritional support compared to patients with low tyrosine levels (HR 0.45 versus 1.46, *p* for interaction = 0.02).

**Conclusion:**

In medical inpatients at nutritional risk, low phenylalanine and tyrosine levels were associated with increased short-and long-term mortality and patients with high tyrosine levels showed a more pronounced response to nutritional support. Further research is warranted to gain a deeper understanding of phenylalanine and tyrosine pathways, their association with clinical outcomes in patients at nutritional risk, as well as their response to nutritional therapy.

**Clinical trial registration:**

www.clinicaltrials.gov, identifier NCT02517476.

## 1 Introduction

Malnutrition affects up to 30% of all hospitalized medical inpatients, and over 45% of elderly hospitalized inpatients (1–3). Patients with malnutrition have an increased risk of infections, sarcopenia, longer hospital stays, reduced quality of life, complications, and increased mortality (4–8). Various studies, including the Effect of early nutritional support on Frailty, Functional Outcomes, and Recovery of malnourished medical inpatients Trial (EFFORT), have demonstrated the benefit of nutritional support. The interventions resulted in improved clinical outcomes such as better quality of life, fewer complications and rehospitalizations, and lower mortality rates for hospitalized patients at nutritional risk (9–13). However, certain groups of malnourished patients, especially the critically ill, did not show benefits from nutritional support (14–16). It remains unclear which specific patient group would experience more pronounced improvements from nutritional intervention, partly because validation studies are lacking. Recent analyses have found blood biomarkers identifying patients who showed increased benefit from nutritional support (15, 17, 18). We aim to find superior predictive biomarkers to support personalized diagnostic and prognostic values of patients at nutritional risk and increase the efficiency of nutritional support. As amino acids and their associated pathways play a crucial role in various metabolic processes and cellular signaling, they may be useful to characterize human metabolism more precisely and provide promising novel targets for exploration.

The essential amino acid phenylalanine and its closely associated non-essential amino acid tyrosine, which is formed from phenylalanine via phenylalanine hydroxylase, are indispensable for protein synthesis und represent two promising avenues for research (19, 20). Phenylalanine is normally obtained through dietary intake. However, during periods of fasting - as in critically ill patients – muscle tissue is the primary source of plasma phenylalanine. Consequently, plasma phenylalanine levels are a surrogate of whole-body proteolysis (19, 21). Previous studies have shown that elevated phenylalanine plasma levels are associated with increased mortality in critically ill patients (22, 23). Furthermore, phenylalanine / tyrosine ratios are known to be abnormal in malnourished or anorexic patients (24, 25). Tyrosine is a hormonal precursor for catecholamines (i.e., dopamine, norepinephrine, and epinephrine) and for thyroid hormones, respectively (26–29). Tyrosine also holds an important role in mediating oxidative stress. During oxidative stress, tyrosine can react with free radicals such as peroxynitrite, leading to its detoxification and production of the by-product nitrotyrosine (30, 31). Nitrotyrosine can therefore be considered a marker for oxidative stress. It remains unclear whether high levels indicate low oxidative stress due to high tyrosine consumption for detoxification or whether the opposite is the case (32). Previous studies in patients with community acquired pneumonia have shown that low plasma nitrotyrosine levels are associated with a lower risk of short-term mortality, whereas in patients with acute kidney injury (AKI), high nitrotyrosine levels and high nitrotyrosine / tyrosine ratios were associated with increased risk of mortality (33, 34). As mentioned above, tyrosine also serves a precursor to catecholamines which are produced via L-3,4-dihydroxyphenylalanine (DOPA) (26). Catecholamines play a key role in modulating the cardiovascular, metabolic, and immune systems. It is also demonstrated that elevated norepinephrine serum levels in COVID-19 patients, and elevated norepinephrine and epinephrine serum levels in patients with severe head injuries are associated with an increased risk of mortality (35, 36).

Phenylalanine and tyrosine levels are known to be influenced by the nutritional status, and oxidative stress can be induced and exacerbated by malnutrition - and vice versa (25, 37–39). In addition to numerous other metabolic effects, catecholamines also impact the gastrointestinal system by regulating the blood flow, affecting gut mobility and nutrition absorption (40). The aim of this study was to investigate the prognostic significance of phenylalanine, tyrosine and their metabolites nitrotyrosine, DOPA, and dopamine in relation to clinical outcomes and the response to nutritional therapy in patients at nutritional risk.

## 2 Materials and methods

### 2.1 Study design and patient population

This study is a secondary analysis of the EFFORT trial; a pragmatic, investigator-initiated, open-label, non-commercial, multicenter, randomized controlled study conducted at eight hospitals in Switzerland (13). The participating centers were both secondary or tertiary care hospitals, such as the University Clinic in Aarau, the University Hospital in Bern, the cantonal hospitals in Lucerne, St. Gallen, Solothurn, Muensterlingen and Baselland, as well as the regional hospital in Lachen.

The hospitalized patients were screened using the Nutritional Risk Screening 2002 (NRS), a validated tool for assessing the risk of malnutrition. Body mass index (BMI), weight loss, food intake, as well as disease severity were taken into account (41). Patients were assigned scores of 0 to 3 based on each risk predictor, with an additional point given if they were 70 years of age or older. Inclusion criteria were: at least 18 years old, NRS total score ≥ 3 points, hospital stay of at least 5 days, and willingness to give informed consent within 48 h after hospital admission. Enrollment occurred between April 1, 2014 and February 28, 2018. Exclusion criteria were: initial hospital admission in intensive care unit or surgical ward, inability to consume oral nutrition, already receiving nutritional support upon admission, having a terminal condition, admission due to anorexia nervosa, acute pancreatitis, acute liver failure, cystic fibrosis, stem cell transplantation, prior gastric bypass surgery, contraindications for nutritional support, and prior inclusion in the trial. For this secondary analysis, we focused on patients from the University Clinic in Aarau with available metabolite samples. The study protocol received approval from the Ethics Committee of Northwest and Central Switzerland (EKNZ) in January 2014 (registration ID 2014_001).

### 2.2 Randomization and procedures

We used secuTrial© software for data collection and management. Patients were randomly allocated to either the control or intervention group. In the intervention group nutritional support was initiated within 48 h of hospital admission, guided by a registered trained dietitian who prescribed individualized nutritional treatment aimed at achieving protein and caloric targets. Nutritional support was not continued after hospital discharge. The nutritional algorithm utilized during the trial is described in detail in the original publication (13). Control group participants received usual hospital meals, with appetite determining the amount of intake. Throughout the study, participants and investigators were aware of the group assignments, however the structured telephone interviews to assess clinical outcomes post-discharge were conducted by blinded study nurses.

### 2.3 Analysis of blood biomarkers

Upon enrollment in the study, blood samples were routinely collected by venipuncture with Vacutrainer Plasma Separator Tubes (Beckton Dickinson, Allschwil, Switzerland). The samples were processed and centrifuged immediately. The plasma was separated and frozen in aliquots and stored at a controlled temperature of-80°C within 2 h after sample collection, with no freeze thaw cycles until further analysis. At least two aliquots of 1 mL for each participant were stored. Plasma metabolites were analyzed between February 2019 and April 2019 using liquid chromatography coupled with tandem mass spectrometry (LC–MS/MS). The analysis utilized an Ultimate 3,000 UHPLC system (Thermo Fisher, San Jose, CA, United States) coupled with a Sciex QTRAP 5500 linear ion-trap quadrupole mass spectrometer (Sciex, Darmstadt, Germany) and the AbsoluteIDQ® p180 kit (BIOCRATES Life Sciences AG, Innsbruck, Austria) (42–44). More detailed, sample preparation was automated on an Evoware 100 (TECAN, Männedorf, Switzerland) following the manufacturer’s protocol. The internal standard solution was dispensed into each well of the provided filter plate by a four-channel liquid handler. Standard, quality control, or participant plasma was added accordingly. After reduction to dryness by a gentle nitrogen stream on an Evaporex (SPT Labtech, Grosslöbichau, Germany), phenylisothiocyanate solution was added. After incubation and another drying step, the metabolites and derivatives were extracted with mass spectrometry-grade methanol and filtered by centrifugation. Before analysis, a final dilution step with water was introduced. Quality controls at three levels provided by the kit manufacturer were run on each plate twice and returned values within the manufacturer’s acceptance range. The analysis kit and calibration solutions were ensured to be from the same lot over the entire period of analysis. A drift in the quality control results was not observed. All plates were analyzed on the same instrument and by the same analyst. The chromatographic front-end underwent planned maintenance and repair right before the analysis. An inter-laboratory evaluation of this commercially available kit for targeted metabolomics demonstrated the reliability of the metabolomics assay (45–47). A total of 238 blood samples of 238 randomly chosen patients recruited in the main study site were analyzed.

### 2.4 Outcomes

The primary endpoint was all-cause mortality within 30 days. Secondary endpoints included mid-and long-term mortality up to 5 years, adverse events within 30 days, major complications such as respiratory failure, major cardiovascular events, acute renal failure, gastro-intestinal failure, decline in functional status measured by the Barthel Index (48), total length of hospital stay, and incidence of falls. These endpoints were assessed via telephone interviews at 30 and 180 days, as well as 2 and 5 years after trial enrollment.

### 2.5 Statistical analysis

#### 2.5.1 Experimental design and patient population of secondary analysis

For the secondary analysis, we assigned patients to groups stratified by their amino acid plasma levels. The Liu method and Receiver Operating Characteristics (ROC) analysis were used to determine the optimal cut points (49). This resulted in the formation of two patient groups per metabolite: low versus high phenylalanine, respectively, tyrosine levels. Of the 238 patients, 125 exhibited phenylalanine levels below the calculated cut point, while 121 had low tyrosine levels. In addition to the tyrosine and phenylalanine blood levels, the phenylalanine to tyrosine ratio and the tyrosine to nitrotyrosine ratio were also calculated. A censoring issue was encountered in the additional analysis of the metabolites nitrotyrosine, DOPA, and dopamine. Specifically, 168 of 238 observations for nitrotyrosine, 101/238 for DOPA, and 177/238 for dopamine fell below the lower limit of detection. Addressing this problem, we stratified patients into three groups: *high* and *low* using the Liu method with ROC of all detectable values, and *very low* for values falling below detection limits.

#### 2.5.2 Statistical analysis

STATA 17.0 was used for the statistical analysis. Continuous variables were expressed as mean ± standard deviation (SD), and binary and categorical variables as number or count and percentages. Statistical significance was tested at 95% confidence intervals (CI), leading to a *p*-value of 0.05. Baseline characteristics were compared between patients with low or high phenylalanine, resp. tyrosine levels; the 2-sample t-test was used for continuous, and the Pearson’s chi-squared test for categorical and binary variables. Logistic and linear regressions were calculated to investigate associations between baseline characteristics and malnutrition parameters with metabolite levels. We specified covariates including sex, baseline nutritional status, Charlson Comorbidity Index (CCI), C-reactive protein (CRP), and randomization group to adjust for potential confounding and random imbalances in regression analyses. To investigate the possible prognostic value for risk of mortality, we assessed metabolite levels associations using Cox regression analysis, calculating Hazard ratios and plotting Kaplan–Meier curves. To analyze the other secondary outcomes, logistic and linear regression models were performed, and odds ratios and coefficient reported. We compared hazard ratios of 30-day all-cause mortality in the intervention versus control groups - stratified by high versus low metabolite levels - to investigate whether response to nutritional support was related to metabolite levels.

## 3 Results

### 3.1 Patient population, baseline characteristics and nutritional parameters

Of the initial 2,088 patient cohort enrolled in the EFFORT trial, complete clinical data including admission metabolite levels was available for 238 patients (11.5%). A total of 116 patients were randomized to the intervention group and 122 to the control group. The study flow chart is illustrated in Supplementary Figure S1. Of the 238 patients, 125 exhibited phenylalanine levels below the calculated cut point (90.45 μmoL/L), whereas 121 had low tyrosine levels (tyrosine cut point 68.65 μmoL/L). The baseline characteristics including nutritional parameters stratified for low and high phenylalanine and tyrosine levels are shown in Table 1. Most baseline characteristics were evenly distributed. However, fewer patients with lower tyrosine plasma levels had a gastrointestinal disease as main diagnosis compared to those with higher tyrosine plasma levels. The risk of malnutrition indicated by the NRS total score was evenly distributed across the groups. Nevertheless, in the low phenylalanine and low tyrosine groups, weight and BMI were notably reduced compared to the high tyrosine and phenylalanine groups (mean weight of patients with high tyrosine 71 kg, compared to low tyrosine 66 kg, *p* = 0.008, mean weight of patients with high phenylalanine 71 kg, compared to low phenylalanine 66 kg, *p* = 0.014). Baseline characteristics and nutritional parameters for nitrotyrosine, DOPA and dopamine were distributed equally (Supplementary Table S1).

**Table 1:** Baseline characteristics stratified by high vs. low tyrosine and phenylalanine.

	Total	High tyrosine	Low tyrosine	*p*-value	High phenylalanine	Low phenylalanine	*p*-value
		> 68.65 μmoL/L	< 68.65 μmoL/L		> 90.45 μmoL/L	< 90.45 μmoL/L	
*n* (%)	*N* = 238	*N* = 117	*N* = 121		*N* = 113	*N* = 125	
Demographic factors							
Male sex	137 (57.6%)	73 (62.4%)	64 (52.9%)	0.14	69 (61.1%)	68 (54.4%)	0.30
Age, mean (SD), years	73.4 (13.5)	73.2 (13.0)	73.6 (14.1)	0.84	72.6 (13.2)	74.0 (13.9)	0.42
Nutritional assessment							
BMI, mean (SD), kg/m^2^	24 (5)	25 (5)	23 (5)	0.009	25 (5)	24 (5)	0.043
Weight, mean (SD), kg	69 (15)	71 (14)	66 (15)	0.008	71 (16)	66 (14)	0.014
Height, mean (SD), cm	168.1 (8.6)	168.6 (8.3)	167.7 (8.9)	0.40	168.9 (8.6)	167.4 (8.5)	0.19
NRS total score				0.80			0.70
3	62 (26.1%)	32 (27.4%)	30 (24.8%)		30 (26.5%)	32 (25.6%)	
4	80 (33.6%)	37 (31.6%)	43 (35.5%)		35 (31.0%)	45 (36.0%)	
5	96 (40.3%)	48 (41.0%)	48 (39.7%)		48 (42.5%)	48 (38.4%)	
Admission main diagnosis							
Infectious disease	65 (27.3%)	29 (24.8%)	36 (29.8%)	0.39	30 (26.5%)	35 (28.0%)	0.80
Cancer disease	75 (31.5%)	35 (29.9%)	40 (33.1%)	0.60	31 (27.4%)	44 (35.2%)	0.20
Cardiovascular disease	24 (10.1%)	11 (9.4%)	13 (10.7%)	0.73	13 (11.5%)	11 (8.8%)	0.49
Frailty	13 (5.5%)	7 (6.0%)	6 (5.0%)	0.73	5 (4.4%)	8 (6.4%)	0.50
Lung disease	11 (4.6%)	6 (5.1%)	5 (4.1%)	0.71	6 (5.3%)	5 (4.0%)	0.63
Gastrointestinal disease	13 (5.5%)	10 (8.5%)	3 (2.5%)	0.039	7 (6.2%)	6 (4.8%)	0.64
Neurological disease	4 (1.7%)	2 (1.7%)	2 (1.7%)	0.97	2 (1.8%)	2 (1.6%)	0.92
Renal disease	15 (6.3%)	9 (7.7%)	6 (5.0%)	0.39	9 (8.0%)	6 (4.8%)	0.32
Metabolic disease	6 (2.5%)	3 (2.6%)	3 (2.5%)	0.97	4 (3.5%)	2 (1.6%)	0.34
Other disease	3 (1.3%)	1 (0.9%)	2 (1.7%)	0.58	2 (1.8%)	1 (0.8%)	0.50
Comorbidities							
Hypertension	139 (58.4%)	73 (62.4%)	66 (54.5%)	0.22	66 (58.4%)	73 (58.4%)	1.00
Malignant disease	113 (47.5%)	54 (46.2%)	59 (48.8%)	0.69	54 (47.8%)	59 (47.2%)	0.93
Chronic kidney disease	81 (34.0%)	45 (38.5%)	36 (29.8%)	0.16	42 (37.2%)	39 (31.2%)	0.33
Coronary heart disease	54 (22.7%)	28 (23.9%)	26 (21.5%)	0.65	28 (24.8%)	26 (20.8%)	0.46
Diabetes mellitus	43 (18.1%)	25 (21.4%)	18 (14.9%)	0.19	23 (20.4%)	20 (16.0%)	0.38
Congestive heart failure	45 (18.9%)	23 (19.7%)	22 (18.2%)	0.77	19 (16.8%)	26 (20.8%)	0.43
COPD	28 (11.8%)	12 (10.3%)	16 (13.2%)	0.48	13 (11.5%)	15 (12.0%)	0.91
Peripheral arterial disease	26 (10.9%)	9 (7.7%)	17 (14.0%)	0.12	11 (9.7%)	15 (12.0%)	0.58
Cerebrovascular disease	27 (11.3%)	13 (11.1%)	14 (11.6%)	0.91	14 (12.4%)	13 (10.4%)	0.63
Dementia	11 (4.6%)	6 (5.1%)	5 (4.1%)	0.71	8 (7.1%)	3 (2.4%)	0.086

BMI, body mass index; COPD, chronic obstructive pulmonary disease; *n*, number; NRS, nutritional risk screening; SD, standard deviation.

Low levels are defined as less than or equal to the cut point value, and high levels are defined as greater than the cut point value.

### 3.2 Associations of phenylalanine and tyrosine with nutritional parameters

No association between nutritional parameters and phenylalanine plasma levels could be observed. Regarding tyrosine, most nutritional parameters showed no significant association; only patients with a BMI >30 kg/m^2^ showed increased tyrosine plasma levels compared to patients with a BMI <18 kg/m^2^ (64.99 μmoL/L versus 79.00 μmoL/L, Coef. 14.01, *p* = 0.02, 95% CI 2.20 to 25.81) (Supplementary Table S2).

### 3.3 Prognostic value of phenylalanine and tyrosine for clinical outcomes

**Figure 1:**
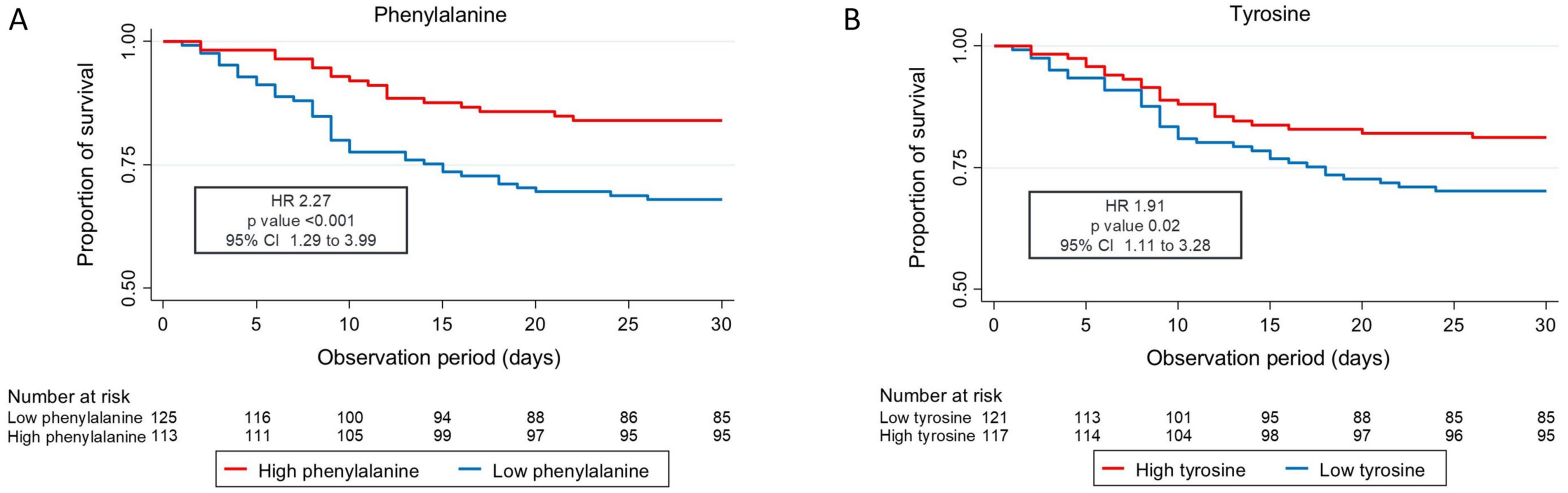
Kaplan Meier curve 30-day all-cause mortality (A) phenylalanine and (B) tyrosine CI, confidence interval; HR, hazard ratio. Low levels are defined as less than or equal to the cut point value, and high levels are defined as greater than the cut point value (cut point tyrosine: 68.65 μmoL/L, cut point phenylalanine: 90.45 μmoL/L). Cox regression analysis.

Low tyrosine and low phenylalanine levels were associated with increased 30-day mortality, persisting even after adjustments were performed: adjusted HR for phenylalanine 2.27 (95% CI 1.29 to 3.00) and adjusted HR for tyrosine 1.91 (95% CI 1.11 to 3.28). These results remained consistent for mid and long-term mortality up to 5 years. Figure 1 shows the probability of survival for 30-day mortality among patients with high versus low phenylalanine and tyrosine plasma levels. Analysis of secondary clinical outcomes found similar trends. Low phenylalanine and tyrosine plasma levels were associated with increased risk of adverse events within 30 days [OR 2.28 (95% CI 1.28 to 4.05)], as well as higher rates of functional decline >10% (Barthel Index) [OR 2.54 (95% CI 1.31 to 4.92)] (Table 2). Full results are presented in Supplementary Table S3. No significant differences in outcome were observed regarding phenylalanine/tyrosine ratio.

**Table 2:** Prognostic value of low and high tyrosine and phenylalanine on mortality rate and adverse events and Barthel Index decline.

	Tyrosine high	Tyrosine low	Adj. HR, OR	95% CI	*p*-value	Phenylalanine high	Phenylalanine low	Adj. HR, OR	95% CI	*p*-value
	*n* (%)	*n* (%)				*n* (%)	*n* (%)			
N	117	121				113	125			
1° Endpoint										
30-day all-cause mortality	22 (18.8%)	36 (29.8%)	1.91	1.11 to 3.28	0.020	18 (15.9%)	40 (32.0%)	2.27	1.29 to 3.99	0.004
2° Endpoint										
180-day all-cause mortality	43 (36.8%)	59 (48.8%)	1.73	1.15 to 2.60	0.009	38 (33.6%)	64 (51.2%)	1.91	1.26 to 2.88	0.002
1-year all-cause mortality	53 (45.3%)	64 (52.9%)	1.52	1.04 to 2.23	0.031	46 (40.7%)	71 (56.8%)	1.76	1.20 to 2.58	0.004
2-year all-cause mortality	65 (55.6%)	74 (61.2%)	1.47	1.04 to 2.09	0.029	61 (54.0%)	78 (62.4%)	1.53	1.08 to 2.16	0.017
3-year all-cause mortality	70 (59.8%)	77 (63.6%)	1.43	1.02 to 2.00	0.039	65 (57.5%)	82 (65.6%)	1.53	1.09 to 2.14	0.014
5-year all-cause mortality	76 (65.0%)	84 (69.4%)	1.43	1.03 to 1.97	0.031	71 (62.8%)	89 (71.2%)	1.47	1.07 to 2.03	0.019
Adverse events within 30 days	36 (30.8%)	57 (47.1%)	2.28	1.28 to 4.05	0.005	33 (29.2%)	60 (48.0%)	2.32	1.31 to 4.11	0.004
Barthel Index decline by >10 points	24 (20.5%)	41 (33.9%)	2.54	1.31 to 4.92	0.006	21 (18.6%)	44 (35.2%)	2.59	1.34 to 5.02	0.005

Adj., Adjusted; CI, confidence interval; HR, hazard ratio; *n*, number; OR, odds ratio.

Cox regression analysis for mortality, logistic regression analysis for adverse events and Barthel Index deline. Adjusted for sex, NRS, intervention-group, CCI, CRP.

### 3.4 Prognostic value of nitrotyrosine, DOPA, and dopamine for clinical outcome

In a further step, we calculated the capacity of nitrotyrosine, DOPA, and dopamine to predict clinical outcomes. Patients were assigned to either *high, low,* or *very low* (below lower limit of detection) metabolite plasma level groups. No significant association with 30-day all-cause mortality was observed for nitrotyrosine; however, we observed a trend toward higher risk of mortality with lower nitrotyrosine levels (nitrotyrosine very low versus high: HR 1.97, 95% CI 0.78 to 4.96). The nitrotyrosine/tyrosine ratio also did not show significant prognostic ability for clinical outcomes. For DOPA and dopamine, a significantly higher risk of mortality within 30 days was associated with high plasma levels; DOPA low versus high: HR 0.31 (95% CI 0.14 to 0.70), dopamine very low versus high: HR 0.031 (95% CI 0.17 to 0.56). Moreover, a reduced risk of mortality over the 5-year period was observed in patients with very low DOPA levels compared to the patients with high DOPA level (HR 0.62, 95% CI 0.42 to 0.92) (Table 3). Kaplan–Meier curves illustrate the risk of 30-day mortality stratified by high, low, and very low metabolite plasma levels (Supplementary Figure S2). Regarding the tyrosine to nitrotyrosine ratio no significant differences in outcome were found.

**Table 3:** Prognostic value of high, low, and very low nitrotyrosine, DOPA, and dopamine on mortality rate.

		30-day all-cause mortality				5-year all-cause mortality			
		*n*/total (%)	HR	*P*-Value	95% CI	*n*/total (%)	HR	*P*-Value	95% CI
Nitrotyrosine									
	High	5 /36 (13.9%)	Reference			24/ 36 (66.7%)	Reference		
	Low	7/34 (20.9%)	1.23	0.734	0.37 to 4.06	26/34 (76.5%)	1.11	0.713	0.63 to 1.95
	Very low	46/168 (27.4%)	1.97	0.151	0.78 to 4.96	110/168 (65.5%)	1.13	0.599	0.72 to 1.77
DOPA									
	High	25/66 (37.9%)	Reference			48/66 (72.7%)	Reference		
	Low	9/71 (12.7%)	0.31	0.005	0.14 to 0.70	51/71 (71.8%)	0.81	0.306	0.54 to 1.22
	Very low	24/101 (23.8%)	0.67	0.169	0.38 to 1.18	61/101 (60.4%)	0.62	0.017	0.42 to 0.92
Dopamine									
	High	15/31 (48.4%)	Reference			23/31 (74.2%)	Reference		
	Low	7/30 (23.3%)	0.30	0.011	0.12 to 0.76	20/30 (66.7%)	0.67	0.212	0.36 to 1.25
	Very low	36/177 (20.3)	0.31	0.000	0.17 to 0.56	117/177 (66.1%)	0.58	0.019	0.37 to 0.92

CI, confidence interval; DOPA, L-3,4-dihydroxyphenylalanine; HR, hazard ratio; *n*, number; OR, odds ratio.

Low levels are defined as less than or equal to the cut point value, high levels are defined as greater than the cut point value, and very low level are defined as not detectable low.

Cox regression analysis, adjusted for sex, NRS, intervention-group, CCI. *n*, number; HR, hazard ratio; CI, confidence interval.

### 3.5 Association of metabolite levels with the respond to nutritional support

**Figure 2:**
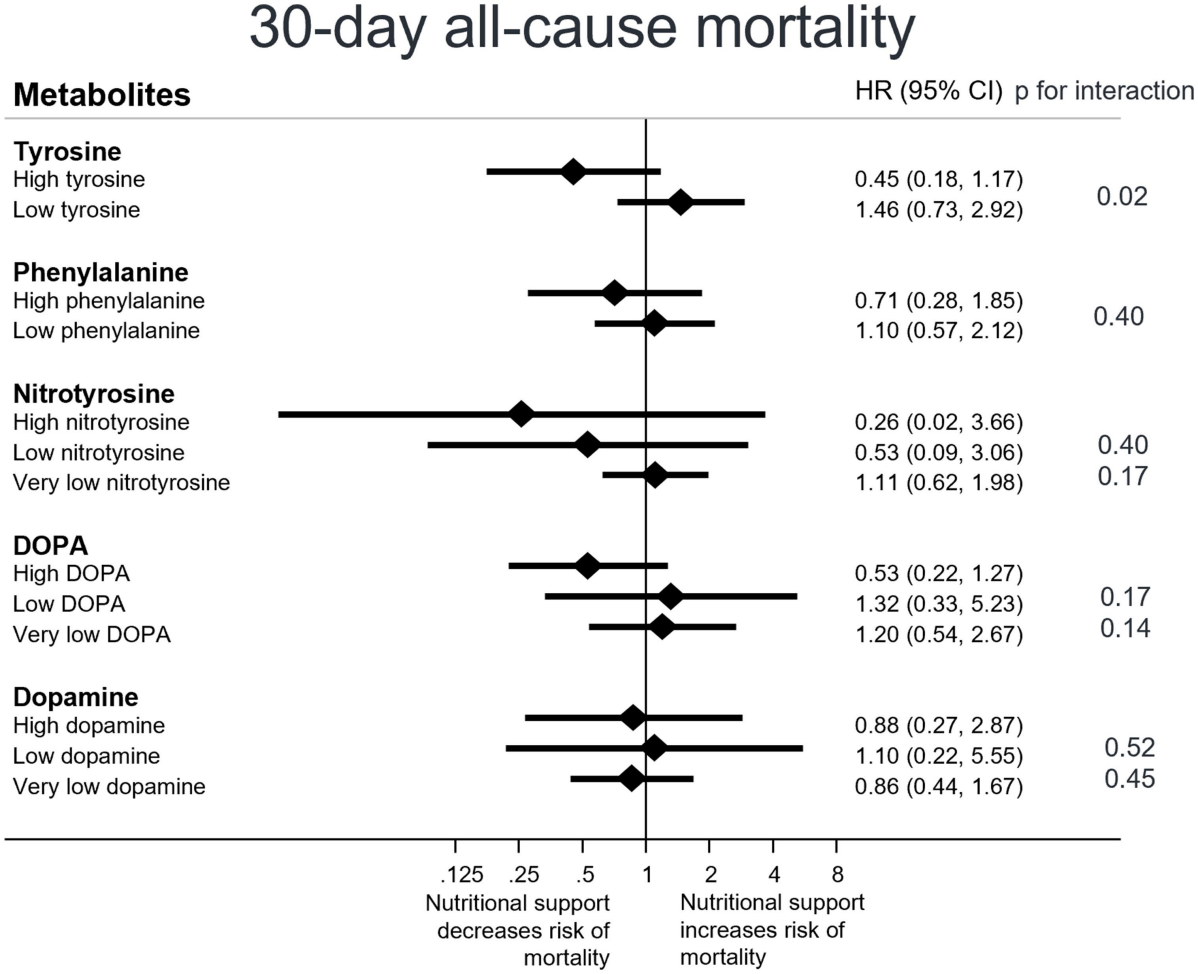
Response to nutritional support on 30-day mortality overall, stratified by patients’ metabolite plasmalevels. CI, confidence interval; HR, hazard ratio Low levels are defined as less than or equal to the cut point value, high levels are defined as greater than the cut point value, and very low levels are defined as values falling below detection limits. Cox regression analysis.

Finally, we examined the impact of nutritional support on 30-day all-cause mortality in patients with high versus low metabolite plasma levels. Patients with high tyrosine levels showed a more pronounced response to nutritional support compared to patients with low tyrosine levels (HR high 0.45, low 1.46, *p* interaction = 0.02); an effect not observed with the other metabolites. For phenylalanine, however, a similar trend of a more pronounced response to nutritional support in patients with high plasma levels versus patients with low plasma levels (HR high 0.71, HR low 1.10, *p* interaction 0.40) was found. Regarding nitrotyrosine and DOPA, trends indicate that patients with high nitrotyrosine (resp. high DOPA) plasma levels may benefit more from nutritional support (Figure 2).

## 4 Discussion

This secondary analysis of the randomized controlled EFFORT trial investigating the value of phenylalanine and tyrosine and its metabolites as prognostic biomarkers among patients at nutritional risk, unveiled several novel findings. Firstly, low levels of phenylalanine and tyrosine were associated with an increased risk of short-and long-term mortality. Secondly, elevated DOPA and dopamine levels were associated with lower short-and long-term survival rates, with a similar trend observed in patients with low nitrotyrosine levels. Finally, our data suggests that although high levels of tyrosine were not linked with increased risk of mortality, these patients show a more pronounced response to nutritional support in terms of 30-day mortality. These findings warrant further discussion.

We found an association between low plasma levels of phenylalanine and tyrosine and an increased risk of short-and long-term mortality. This indicates that phenylalanine and tyrosine plasma levels provided prognostic information in our patient cohort. However, no associations between plasma levels and the NRS or disease severity were observed. Nevertheless, low phenylalanine and tyrosine plasma levels correlated with lower weight and BMI. Interestingly, mean phenylalanine and tyrosine plasma levels in our cohort were slightly elevated compared to those of a healthy French cohort (45). These findings contradicted our expectations that malnutrition leads to decreased amino acid levels (phenylalanine mean French cohort: 60.0 ± 7.75umol/l, phenylalanine mean EFFORT cohort: 91.96umol/l, tyrosine mean French cohort: 62.9 ± 12.2umol/l, tyrosine mean EFFORT cohort: 69.82umol/l) (45). We attribute the elevated levels to pronounced oxidative stress experience by our patients due to acute illness and malnutrition. Oxidative stress can induce hyperphenylalaninemia and elevate tyrosine plasma levels through various pathways - including muscle catabolism and whole-body proteolysis - resulting in the release of amino acids into circulation (50, 51). In contrast to findings in other studies where high phenylalanine plasma levels were associated with increased morbidity and mortality, our analysis reveals that lower plasma phenylalanine and tyrosine levels predicted worse clinical outcomes; with phenylalanine possessing the strongest prognostic value (22, 23, 52–55). Several mechanisms might contribute to these differences. These include impaired adaptation to oxidative stress; whereby lower phenylalanine plasma levels lead to reduced tyrosine plasma levels and result in diminished detoxification of peroxynitrite. Additionally, decreased protein synthesis due to insufficient building blocks may also underlie these associations (4, 56). Another notable difference between the above studies is that the populations studied are different. For example, in a multicenter validation in critically ill patients, Tsou et al. found that very high phenylalanine levels were most strongly associated with mortality (22). These levels were significantly higher than in our non-critically ill population, suggesting a U-shaped relationship between phenylalanine and mortality, with non-critically ill patients unlikely to reach the very high levels. A hypothesis that is also consistent with observations of other amino acids (57). Regarding tyrosine, we also hypothesize an association between increased mortality in patients with low T3 syndrome and the increased mortality observed in our patients with reduced tyrosine levels (58). This because tyrosine serves as a precursor for thyroid hormones, and its plasma levels are directly associated with fT3 plasma levels (59, 60). As tyrosine not only serves as a precursor for thyroid hormones, but also for catecholamines, we conducted a more detailed analysis of its metabolites DOPA and dopamine. We observed associations between high DOPA and dopamine levels with increased risk of mortality. Given that DOPA and dopamine are direct precursors of catecholamines, our findings align with previous studies which reported links between elevated norepinephrine and epinephrine plasma levels and increased mortality in patients with COVID-19, severe head injury, and community acquired pneumonia (35, 36). Catecholamines effect the hypothalamic, pituitary, and adrenal axis influencing cortisol production, and release and play a role in the regulation of various physiological processes. Elevated catecholamine levels may contribute to cardiovascular dysfunction, hypercoagulability, pulmonary and immune dysregulation, as well as diabetogenic states (35). Our findings add further insight into the metabolism and pathophysiological role of catecholamines and their precursors. Another novel trend indicates that low nitrotyrosine levels may be associated with increased risk of mortality, contradicts a study demonstrating that low nitrotyrosine levels are associated with a lower mortality rate in patients with CAP (34). Since our patient cohort had enhanced oxidative stress due to acute illness and malnutrition, elevated levels of nitrotyrosine could indicate successful detoxification of the toxic radical peroxynitrite by tyrosine. Conversely, low levels of nitrotyrosine might indicate inadequate coping with oxidative stress and a failure to detoxify toxic oxygen radicals, which in turn could potentially increase the risk of mortality. In addition to metabolite values, we also analyzed the phenylalanine to tyrosine ratio and the tyrosine to nitrotyrosine ratio. It is known that the phenylalanine to tyrosine ratio tends to be abnormal in malnourished and anorectic patients, and previous studies have shown that a higher tyrosine to nitrotyrosine ratio can predict a higher risk of mortality in patients with community-acquired pneumonia (19, 33, 34, 61). However, in our analysis, none of the ratios showed any differences regarding mortality, severity of malnutrition or response to nutritional support. Our results may differ because the participants were not anorexic, as anorexia nervosa was an exclusion criterion of the EFFORT trail, and only 27.3% had an infectious disease. Furthermore, our cohort was a rather older, more polymorbid and heterogenous patient population with a variety of main diagnoses compared to the studies of the ratios. Therefore, we cannot confirm the prognostic value of investigated ratios in our cohorts. As the metabolic pathways and processes involved are highly complex and influenced by various factors, our findings and explanations should be viewed as highly exploratory and somewhat speculative. The absence of normative values for DOPA, dopamine, and nitrotyrosine plasma levels also make our findings difficult to interpret.

A final novel finding of this analysis was that although low tyrosine levels were associated with an increased risk of mortality, lower BMI and lower weight, nutritional support was significantly less effective in improving outcomes compared to patients with higher tyrosine plasma levels. Patients with higher tyrosine levels showed a more pronounced response. Although low plasma levels of tyrosine were not associated with disease severity assessed via NRS score, we believe our findings to be in line with studies of critically ill patients who gained minimal benefit from full-replacement feeding (14, 16). High nutritional intake in the critically ill has been proposed to reduce autophagy, which is crucial for cell detoxification and may explain the lack of effect observed in these patients (62). If validated in larger prospective analyses, our findings support the testing of these metabolites to explain variability in response to treatment and - together with other scores - to further personalize nutritional support.

Concurrently, tyrosine supplementation has been proposed to enhance cognition, but not enhance the ability for physical exercises (63), although other trials have shown that additional tyrosine supplementation was associated with increased exercise capacity in hot environments (64). To our knowledge there are no studies answering the question of whether tyrosine supplementation impacts survival rates.

### 4.1 Strength and limitations

This report has both strengths and limitations. We present the first investigation of correlations between phenylalanine, tyrosine, and metabolites with clinical outcome and response to nutritional support among patient at risk of malnutrition. We possess data from a robust randomized controlled trial with a well-characterized patient cohort and prospectively collected short-and long-term outcomes measures. This allowed us to adjust for various confounding variables. Our study had a focused research question; therefore, we did not adjust for multiple comparisons. As this is a secondary analysis, results should be interpreted as exploratory and hypothesis generating. Our analysis was limited to a subgroup from one center with a smaller patient population, which resulted in reduced power and limited external validity. Furthermore, we encountered limitations related to the metabolomic kit used, as it has been primarily applied for research purposes and lacks well validated reference values. We also faced a censoring issue wherein some patient’s plasma levels of nitrotyrosine, DOPA and dopamine fell below the lower level of detection. This necessitated organizing our patient cohort into three groups based on the plasma levels (high, low and below the limit of detection), making results more vulnerable to incorrect assumptions. Finally, as amino acid and metabolite plasma levels were only measured upon study inclusion, we were unable to assess the influence of nutritional therapy on these metabolites.

## 5 Conclusion

This secondary analysis of the EFFORT trial revealed that among medical inpatients at nutritional risk, low phenylalanine and tyrosine levels were associated with increased short-and long-term mortality and adverse outcomes, making these amino acids independent prognostic markers for this patient population. Furthermore, DOPA and dopamine plasma levels may also serve as prognostic markers for mortality. Lastly, patients with high tyrosine levels showed a more pronounced response to nutritional support compared to those with low tyrosine levels. Further research is warranted to understand how phenylalanine and tyrosine pathways influence, and could potentially improve, the nutritional management of patients.

## Data Availability

The data analyzed in this study are subject to the following licenses/restrictions: Data described in the manuscript, codebook, and analytic code are available upon request after other secondary projects related to the study are completed. Requests to access these datasets should be directed to philipp.schuetz@unibas.ch.
