# Supplementary material for: Association of phenylalanine and tyrosine metabolism with mortality and response to nutritional support among patients at nutritional risk: a secondary analysis of the randomized clinical trial EFFORT
Source: Front Nutr. 2024 Nov 12;11:1451081. doi: 10.3389/fnut.2024.1451081 (PMC11588475; doi:10.3389/fnut.2024.1451081)
Supplement: Supplementary file 1 [file Data_Sheet_1.PDF]

Supplementary Material

Association of phenylalanine and tyrosine metabolism with mortality and response to nutritional support among patients at nutritional risk - A secondary analysis of the randomized clinical trial EFFORT

Supplemental Table 1 Baseline characteristics and nutritional parameters stratified by high, low, very low nitrotyrosine, DOPA, and dopamine

		Nitrotyrosine				DOPA				Dopamine			
	Total	high	low	very low	p-value	high	low	very low	p-value	high	low	very low	p-value
n (%)	N=238	N=36	N=34	N=168		N=66	N=71	N=101		N=36	N=30	N=177	
**Demografic factors**													
Male sex	137 (57.6%)	18 (50.0%)	18 (52.9%)	101 (60.1%)	0.45	44 (66.7%)	36 (50.7%)	57 (56.4%)	0.16	18 (50.0%)	17 (56.7%)	101 (57.1%)	0.90
Age, mean (SD), years	73.4 (13.5)	72.9 (12.1)	79.4 (9.6)	72.3 (14.3)	**0.019**	72.4 (12.1)	74.5 (13.1)	73.2 (14.8)	0.64	72.9 (12.1)	71.1 (11.7)	73.8 (14.0)	0.59
**Nutritional assessment**													
BMI, mean (SD), kg/m²	24 (5)	25 (5)	24 (5)	24 (5)	0.85	24 (4)	25 (5)	24 (5)	0.35	25 (5)	25 (4)	24 (5)	0.72
Weight, mean (SD), kg	69 (15)	70 (14)	68 (15)	69 (15)	0.90	69 (15)	70 (15)	67 (15)	0.44	70 (14)	70 (14)	69 (15)	0.92
Height, mean (SD), cm	168.1 (8.6)	168.4 (9.7)	166.6 (8.6)	168.4 (8.4)	0.54	168.3 (8.7)	167.9 (10.1)	168.2 (7.3)	0.96	168.4 (9.7)	167.3 (8.1)	168.1 (8.6)	0.78
NRS total score					0.64				0.50				0.41
3	62 (26.1%)	11 (30.6%)	8 (23.5%)	43 (25.6%)		16 (24.2%)	23 (32.4%)	23 (22.8%)		11 (30.6%)	6 (20.0%)	47 (26.6%)	
4	80 (33.6%)	10 (27.8%)	15 (44.1%)	55 (32.7%)		25 (37.9%)	23 (32.4%)	32 (31.7%)		10 (27.8%)	14 (46.7%)	54 (30.5%)	
5	96 (40.3%)	15 (41.7%)	11 (32.4%)	70 (41.7%)		25 (37.9%)	25 (35.2%)	46 (45.5%)		15 (41.7%)	10 (33.3%)	76 (42.9%)	
**Main diagnosis**													
Infection	65 (27.3%)	6 (16.7%)	8 (23.5%)	51 (30.4%)	0.21	19 (28.8%)	17 (23.9%)	29 (28.7%)	0.75	6 (16.7%)	10 (33.3%)	45 (25.4%)	0.54
Cancer	75 (31.5%)	11 (30.6%)	9 (26.5%)	55 (32.7%)	0.77	26 (39.4%)	17 (23.9%)	32 (31.7%)	0.15	11 (30.6%)	11 (36.7%)	53 (29.9%)	0.67
Cardiovascular disease	24 (10.1%)	6 (16.7%)	5 (14.7%)	13 (7.7%)	0.17	4 (6.1%)	11 (15.5%)	9 (8.9%)	0.16	6 (16.7%)	1 (3.3%)	21 (11.9%)	0.28
Frailty	13 (5.5%)	1 (2.8%)	0 (0.0%)	12 (7.1%)	0.18	4 (6.1%)	1 (1.4%)	8 (7.9%)	0.17	1 (2.8%)	2 (6.7%)	10 (5.6%)	0.82
Lung disease	11 (4.6%)	1 (2.8%)	4 (11.8%)	6 (3.6%)	0.099	3 (4.5%)	4 (5.6%)	4 (4.0%)	0.88	1 (2.8%)	0 (0.0%)	10 (5.6%)	0.37
Gastrointestinal disease	13 (5.5%)	5 (13.9%)	1 (2.9%)	7 (4.2%)	0.052	2 (3.0%)	8 (11.3%)	3 (3.0%)	**0.037**	5 (13.9%)	2 (6.7%)	11 (6.2%)	0.36
Neurological disease	4 (1.7%)	0 (0.0%)	1 (2.9%)	3 (1.8%)	0.62	0 (0.0%)	2 (2.8%)	2 (2.0%)	0.42	0 (0.0%)	0 (0.0%)	4 (2.3%)	0.50
Renal disease	15 (6.3%)	1 (2.8%)	3 (8.8%)	11 (6.5%)	0.57	4 (6.1%)	3 (4.2%)	8 (7.9%)	0.61	1 (2.8%)	3 (10.0%)	9 (5.1%)	0.42
Metabolic disease	6 (2.5%)	2 (5.6%)	1 (2.9%)	3 (1.8%)	0.42	2 (3.0%)	3 (4.2%)	1 (1.0%)	0.39	2 (5.6%)	0 (0.0%)	4 (2.3%)	0.25
Other	3 (1.3%)	1 (2.8%)	0 (0.0%)	2 (1.2%)	0.58	0 (0.0%)	1 (1.4%)	2 (2.0%)	0.53	0 (0.0%)	0 (0.0%)	3 (1.7%)	0.59
Hypertension	139 (58.4%)	23 (63.9%)	19 (55.9%)	97 (57.7%)	0.75	33 (50.0%)	45 (63.4%)	61 (60.4%)	0.25	18 (58.1%)	17 (56.7%)	104 (58.8%)	0.98
Malignant disease	113 (47.5%)	16 (44.4%)	12 (35.3%)	85 (50.6%)	0.25	35 (53.0%)	30 (42.3%)	48 (47.5%)	0.45	15 (48.4%)	19 (63.3%)	79 (44.6%)	0.16
Chronic kidney disease	81 (34.0%)	13 (36.1%)	11 (32.4%)	57 (33.9%)	0.95	24 (36.4%)	23 (32.4%)	34 (33.7%)	0.88	14 (45.2%)	12 (40.0%)	55 (31.1%)	0.24
Coronary heart disease	54 (22.7%)	10 (27.8%)	12 (35.3%)	32 (19.0%)	0.087	14 (21.2%)	18 (25.4%)	22 (21.8%)	0.81	9 (29.0%)	3 (10.0%)	42 (23.7%)	0.17
Diabetes	43 (18.1%)	7 (19.4%)	5 (14.7%)	31 (18.5%)	0.85	12 (18.2%)	12 (16.9%)	19 (18.8%)	0.95	5 (16.1%)	7 (23.3%)	31 (17.5%)	0.71
Congestive heart failure	45 (18.9%)	8 (22.2%)	11 (32.4%)	26 (15.5%)	0.062	11 (16.7%)	19 (26.8%)	15 (14.9%)	0.13	5 (16.1%)	5 (16.7%)	35 (19.8%)	0.84
COPD	28 (11.8%)	2 (5.6%)	6 (17.6%)	20 (11.9%)	0.29	6 (9.1%)	10 (14.1%)	12 (11.9%)	0.66	4 (12.9%)	1 (3.3%)	23 (13.0%)	0.31
Peripheral arterial disease	26 (10.9%)	6 (16.7%)	4 (11.8%)	16 (9.5%)	0.45	5 (7.6%)	10 (14.1%)	11 (10.9%)	0.47	2 (6.5%)	2 (6.7%)	22 (12.4%)	0.45
Cerebrovascular disease	27 (11.3%)	3 (8.3%)	7 (20.6%)	17 (10.1%)	0.18	7 (10.6%)	11 (15.5%)	9 (8.9%)	0.40	4 (12.9%)	2 (6.7%)	21 (11.9%)	0.68
Dementia	11 (4.6%)	2 (5.6%)	4 (11.8%)	5 (3.0%)	0.081	3 (4.5%)	4 (5.6%)	4 (4.0%)	0.88	2 (6.5%)	1 (3.3%)	8 (4.5%)	0.84

N, number; BMI, body mass index; SD, standard deviation; NRS, Nutritional Risk Screening,

Supplemental Table 2 NRS 2022 score stratified by tyrosine and phenylalanine serum levels

	Tyrosine				Phenylalanine			
	Mean	Coefficient	p-value	95%-CI	Mean	Coefficient	p-value	95%-CI
NRS total score								
NRS=3	72.20	reference			93.93	reference		
NRS=4	68.52	-3.69	0.34	-11.35 to 3.97	89.24	-4.68	0.30	-13.60 to 4.23
NRS≥5	69.38	-2.83	0.45	-10.20 to 4.55	92.97	-0.96	0.83	-9.54 to 7.62
Food intake								
>75%	73.82	reference			94.48	reference		
50-75%	69.52	-4.30	0.29	-12.31 to 3.71	93.70	-0.78	0.87	-10.08 to 8.53
25-50%	66.35	-7.47	0.09	-16.14 to 1.19	85.99	-8.49	0.10	-18.55 to 1.57
0-25%	73.01	-0.81	0.89	-12.17 to 10.54	96.23	1.76	0.79	-11.43 to 14.95
BMI, mean, kg/m^2								
<18.5	64.99	reference			93.64	reference		
18.5 - 25	66.74	1.75	0.74	-8.56 to 12.05	88.30	-5.33	0.39	-17.42 to 6.75
25 - 30	71.88	6.89	0.23	-4.33 to 18.10	92.88	-0.75	0.91	-13.91 to 12.40
>30	79.00	14.01	**0.02**	2.20 to 25.81	100.88	7.24	0.30	-6.60 to 21.08
Weight loss								
none	70.23	reference			92.52	reference		
>5% in 3 Mt.	73.07	2.84	0.49	-5.31 to 10.98	93.23	0.72	0.88	-8.78 to 10.22
>5% in 2 Mt.	69.48	-0.75	0.86	-9.19 to 7.69	93.96	1.45	0.77	-8.40 to 11.29
>5% in 1 Mt.	66.20	-4.03	0.32	-11.92 to 3.86	87.88	-4.63	0.32	-13.84 to 4.57
Disease severity								
none	79.40	reference			111.17	reference		
mild	70.71	-8.69	0.52	-35.10 to 17.73	91.70	-19.46	0.21	-50.18 to 11.25
moderate	67.85	-11.55	0.39	-38.17 to 15.08	92.01	-19.16	0.22	-50.12 to 11.80
severe	71.00	-8.40	0.75	-60.71 to 43.91	70.90	-40.27	0.19	-101.09 to 20.56

NRS, Nutritional Risk Screening; BMI, body mass index; CI, confidence interval

Supplemental Table 3 Secondary endpoints, stratified by tyrosine and phenylalanine serum levels

	Coefficient/OR	p-value	95% CI
Adverse event 30 days			
Tyrosin			
continuous	0.989	0.084	0.98 to 1.00
Tyr < cutpoint	2.281	**0.005**	1.28 to 4.05
Phenylalanin			
continuous	0.990	0.065	0.98 to 1.00
Phe < cutpoint	2.321	**0.004**	1.31 to 4.11
ICU 30 days			
Tyrosin			
continuous	1.014	0.514	0.97 to 1.06
Tyr < cutpoint	0.826	0.854	0.11 to 6.31
Phenylalanin			
continuous	0.983	0.477	0.94 to 1.03
Phe < cutpoint	2.888	0.368	0.29 to 29.02
Rehospitalisation 30 days			
Tyrosin			
continuous	0.999	0.932	0.98 to 1.02
Tyr < cutpoint	1.344	0.546	0.51 to 3.51
Phenylalanin			
continuous	0.994	0.548	0.98 to 1.01
Phe < cutpoint	1.648	0.316	0.62 to 4.38
Major Complications 30 days			
Tyrosin			
continuous	1.001	0.908	0.91 to 3.43
Tyr < cutpoint	1.136	0.779	0.47 to 2.78
Phenylalanin			
continuous	0.990	0.302	0.97 to 1.01
Phe < cutpoint	1.636	0.294	0.65 to 4.10
Barthel Decline >10			
Tyrosin			
continuous	0.989	0.143	0.97 to 1.00
Tyr < cutpoint	2.535	**0.006**	1.31 to 4.92
Phenylalanin			
continuous	0.985	0.024	0.97 to 1.00
Phe < cutpoint	2.594	**0.005**	1.34 to 5.02
Falls 180 days			
Tyrosin			
continuous	1.002	0.879	0.98 to 1.02
Tyr < cutpoint	0.949	0.907	0.39 to 2.29
Phenylalanin			
continuous	1.008	0.291	0.99 to 1.02
Phe < cutpoint	0.489	0.119	0.20 to 1.20

Tyr, tyrosine; Phe, phenylalanine; ICU, intensive care unit; OR, Odds ratio; CI; confidence interval

Supplemental Figure 1 Study flow chart of the secondary analysis based on Schuetz et al., 2019

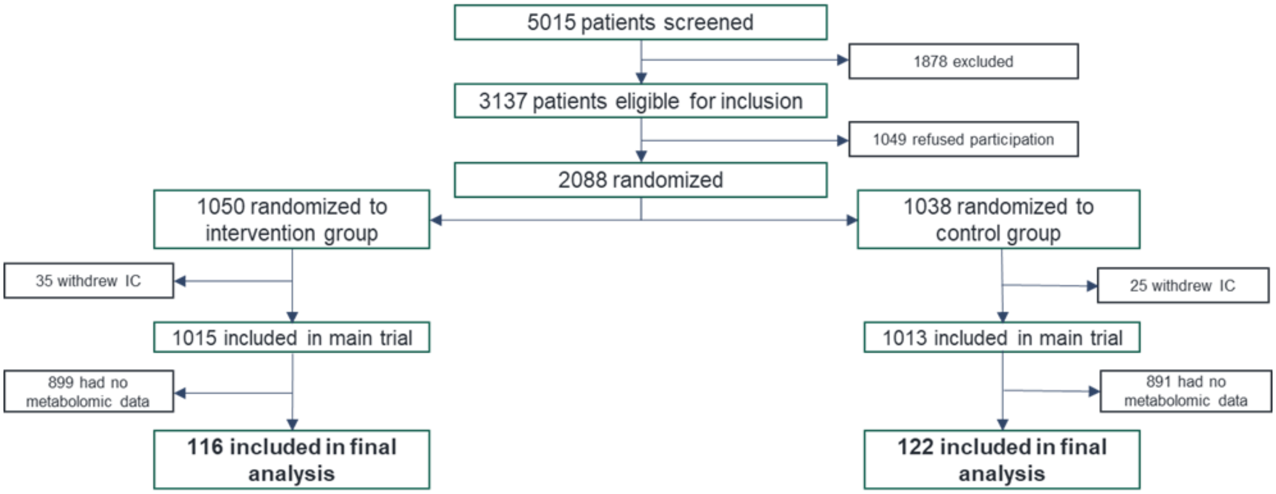

IC, informed consent.

Reasons for exclusion: 145 surgical patients, 268 unable to ingest oral nutrition, 158 terminal condition, 719 already receiving nutritional therapy, 31 anorexia nervosa, 161 acute pancreatitis, 81 acute liver failure, 6 cystic fibrosis, 11 stem-cell transplantation, 27 post gastric bypass operation, 43 contraindications against nutritional support, 228 earlier inclusion.

Supplemental Figure 2 Kaplan Meier curve 30-day all-cause mortality A) nitrotyrosine, B) DOPA, C) dopamine

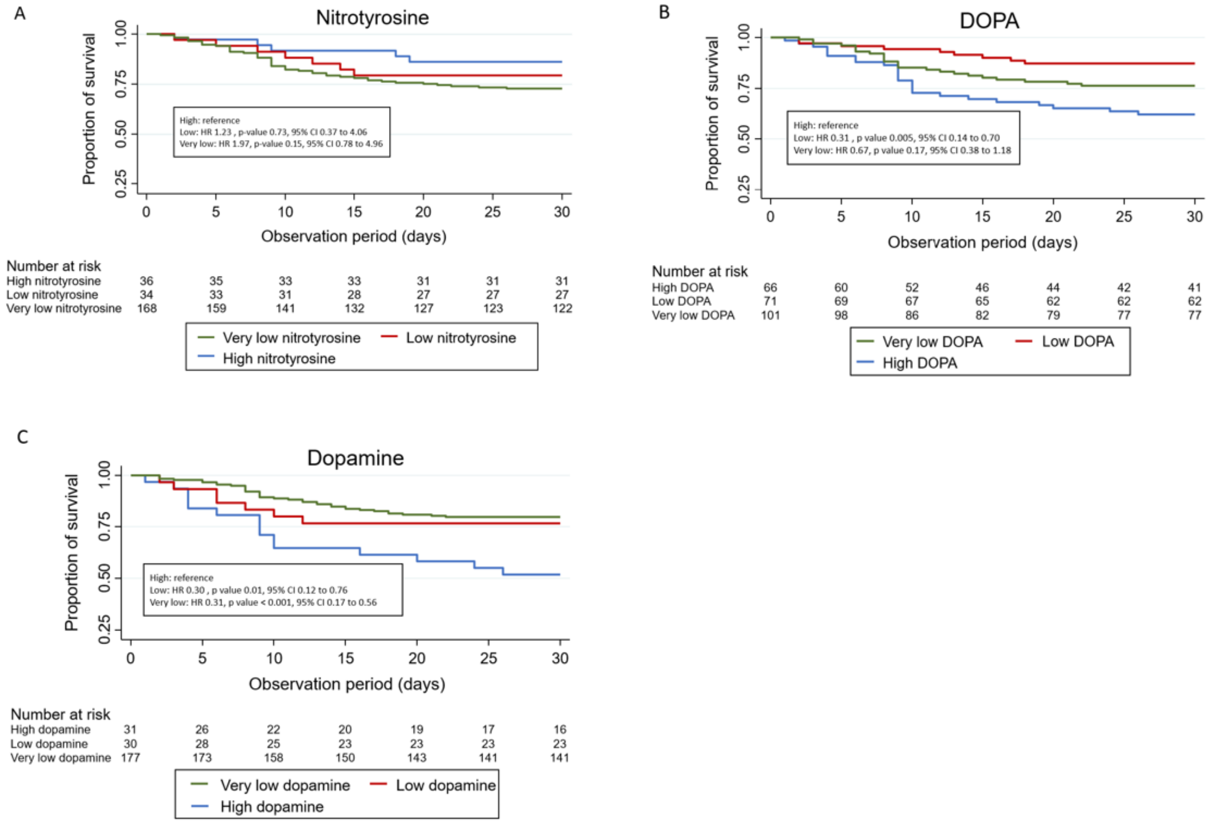

CI, confidence interval; HR, hazard ratio

Low levels are defined as less than or equal to the cut point value, high levels are defined as greater than the cut point value, and very low levels are defined as values falling below detection limits. Cox regression analysis.
